# Novel Application of Voltammetric Sensors to Characterize the Electrochemical Behavior of Porous Media

**DOI:** 10.1021/acsomega.5c12094

**Published:** 2026-02-10

**Authors:** Ana Martínez-Ibernón, José M. Gandía Romero, Josep R. Lliso Ferrando, Isabel Gasch

**Affiliations:** Interuniversity Research Institute for Molecular Recognition and Technological Development (IDM), 16774 Universitat Politècnica de València, Universitat de València, 46022 Valencia, Spain

## Abstract

The objective of this study is to assess the feasibility of using embedded voltammetric sensors for the electrochemical characterization of porous materials, specifically concrete, and to analyze how environmental conditions influence their response. Cyclic voltammograms were recorded in solutions simulating various pore-solution conditions of concrete as well as in concrete specimens under different states (water-saturated, carbonated, and chloride-contaminated), using a gold electrode as the sensing element. The morphology of the voltammograms and characteristic electrochemical parameters (peak current and potential, Δ*E*_p_, *i*_B_/*i*_b_, α*n*, *R*_u_) were examined to identify similarities and differences in the sensor’s behavior across both environments. Subsequently, principal component analysis (PCA) was applied to evaluate the reproducibility of the response and the sensor’s discriminating capability in a porous medium such as concrete. The results indicate that the identified redox process, associated with OH^–^ adsorption/desorption, preserves its electrochemical nature in both environments; however, in concrete, the control mechanism shifts from quasi-reversible to diffusion-kinetic, governed by ionic resistance and the tortuosity of the pore network. The PCA results demonstrate that the embedded sensor effectively differentiates between reference, carbonated, and chloride-contaminated concrete, confirming its potential as a nondestructive diagnostic tool for in situ electrochemical monitoring of porous materials containing interstitial moisture.

## Graphical abstract

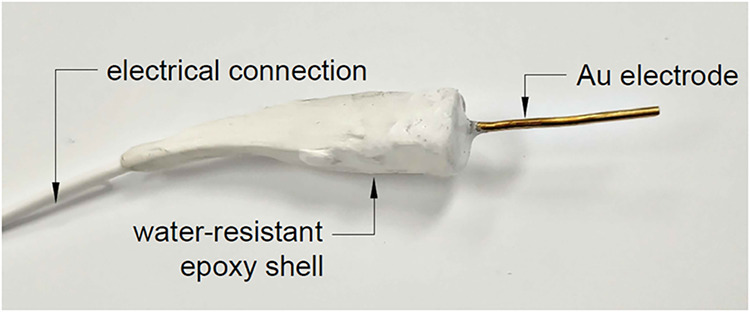

## 1 Introduction

Voltammetric sensors have traditionally been employed in electrochemistry for the analysis and characterization of liquid-phase systems. They are now widely applied across diverse areas involving solution-based characterization and quality control, including wastewater treatment monitoring, food technology, and medical sample analysis.^1–8^ In these fields, the high efficiency and analytical reliability of voltammetric sensors are well established. Current research continues to advance this technology and explore its implementation in new application domains, although predominantly within liquid environments.

In such systems, the basic instrumentation consists of a potentiostat and an electrochemical cell, which, in its most conventional configuration, comprises three electrodes: the working electrode (or sensing electrode, WE), the counter electrode (CE), and the reference electrode (REF).

Advances in instrumentation have focused on making potentiostats lighter and more autonomous, since, due to the bulky nature of traditional equipment, voltammetry has historically been a laboratory-based technique. In recent years, in situ voltammetry has become increasingly common, as the size and cost of potentiometric equipment have been significantly reduced. Commercial products currently available for in situ voltammetric analysis include the μStat 400 bipotentiostat/galvanostat (Metrohm) and pocketSTAT2 (IVIUM Technologies).

Regarding the electrodes employed, microelectrodes, macroelectrodes, and screen-printed electrodes made of various materials have been used. Over the past decade, most studies have focused on enhancing sensor durability and reducing production costs, primarily through the development of thick-film and screen-printed sensors.^9–11^ One of the most recent works in this field is Tyszczuk-Rotko et al.,^12^ which proposed the use of low-cost, screen-printed voltammetric sensors for the detection of analgesic residues in environmental waters. More recently, efforts aimed at further reducing costs and simplifying the fabrication of such sensors, to enable large-scale production, have focused on the application of 3D printing technology.^13–16^

Voltammetric sensors operate using voltammetric techniques in which the excitation signal varies depending on the waveform applied (square wave, differential pulse, or linear sweep). Although the triangular sweep has traditionally been employed, recent studies, such as that by Ramón et al.,^17^ have demonstrated that potentiodynamic pulses reduce electrode alteration without compromising response reliability. Another study aiming to improve sensor performance through excitation signal optimization was conducted by Aiassa et al.,^18^ who applied staircase cyclic voltammetry or differential pulse voltammetry at low sampling frequencies to enhance the efficiency of pharmaceutical detection in aqueous samples.

In addition to the previously mentioned advances, several studies have also focused on improving the operational autonomy and data-processing capabilities of voltammetric sensor systems. For example, Liu et al.^19^ analyzed the benefits of implementing machine learning and deep learning algorithms to enhance the performance of voltammetric sensing platforms.

However, the application of such sensors in media other than liquids has been scarcely explored. Among the research areas with significant untapped potential is the use of embedded voltammetric sensors in porous materials, such as soils or concrete, where the presence of interstitial moisture enables ionic conduction and, consequently, the use of electrochemical sensors. In fact, in porous media such as concrete, other types of electrochemical sensors, including potentiometric,^9,20–24^ galvanic^25–29^ and conductometric sensors,^30,31^ are commonly employed.

When voltammetric sensors are compared with other types of electrochemical sensors that have been used in porous media, voltammetric sensors offer a reduced susceptibility to interference and reaction overlap. For instance, in the case of potentiometric sensors, which are more widely employed, an ion-selective electrode is used to measure potential variation over time with respect to a reference electrode. The potential response follows a Nernstian relationship with the target ion; however, selectivity does not imply exclusivity. These sensors can still be affected by other species present in the medium as well as by variations in temperature, pH, and humidity, often leading to measurement errors caused by interference or overlapping effects. Moreover, their long-term durability cannot be guaranteed.

In contrast, in voltammetric sensors, the application of an excitation signal allows for the promotion of specific electrochemical reactions depending on the applied potential. By analyzing only the portion of the response corresponding to the reaction of interest, interference effects can be significantly minimized. Additionally, techniques such as electropolishing,^1^ which cannot be implemented in potentiometric systems, contribute to enhanced durability by maintaining the sensor surface in a relatively stable condition over time.

Compared with sensors based on other technologies, such as fiber-optic,^32–34^ and piezoelectric sensors,^11,35,36^ which have already been applied in porous materials,^37–41^ voltammetric sensors, as electrochemical devices, offer the advantage that their response is directly related to the composition or concentration of a specific electroactive species or to the presence of a given element or ion.

Another key advantage of voltammetric sensors over all of the aforementioned types is the wealth of information contained in their response signals, which can be processed using multivariate statistical analysis methods such as principal component analysis (PCA), partial least-squares regression (PLS), and artificial neural networks (ANNs).^42–46^

However, these sensors also present certain disadvantages. The quality of the measurement strongly depends on the stability of the reference electrode, and stable reference sensors are often relatively expensive. This limitation can be addressed using a two-electrode cell configuration. Previous studies have shown that when the counter electrode has a surface area at least 40 times larger than that of the working electrode, its behavior can approximate that of a pseudoreference electrode.^47,48^

The application of voltammetric sensors in porous media such as concrete presents some challenges but remains highly promising, as it can enable more reliable and durable monitoring of the condition of porous materials compared to other sensing systems. In the specific case of concrete, integrating this technology into structural elements could represent a key step toward improved durability control and contribute significantly to the development of smart structures for smart cities.

Among the few reported examples of voltammetric sensors embedded in porous materials is the Corrochip system.^49–51^ In this system, a voltammetric sensor is embedded in concrete and subjected to a series of potentiodynamic pulses to determine the corrosion rate by analogy with the Tafel method. Other examples found in the literature, although still based on preliminary results, include the sensor proposed by Bujes-Garrido et al.^50,51^ for chloride detection in concrete, and that presented by Correia et al.^52^ for estimating oxygen availability.

The objective of this work is to demonstrate the potential of voltammetric sensors for the investigation and characterization of porous materials. A three-electrode cell configuration was employed, using a gold electrode as the working electrode, a stainless steel plate as the counter electrode, and a saturated calomel electrode as the reference. The porous material used was medium-porosity concrete. At the same time, this study aims to encourage further research into the use of voltammetric sensors across new fields of application.

### 1.1 Au Electrode

Noble metals such as gold and platinum are extensively employed in electrochemical systems due to their excellent electrocatalytic properties and relative inertness under anodic potentials in both acidic and alkaline electrolytes. Gold, regarded as one of the most noble metals because of its low reactivity,^53^ is an inner transition metal. Its electronic configuration provides a potential range in which the only current recorded arises from the transient charging and discharging of the electrical double layer.^53^ This characteristic facilitates the identification of the ion adsorption and desorption processes in electrochemical experiments conducted within the double-layer potential range. Therefore, gold is highly suitable for analyzing the presence of various ions in electrolytes.^53–56^ Gold electrodes are also essential for investigating the early stages of corrosion mechanisms and kinetics in metals.^57^ Moreover, gold is the only noble metal in which the adsorption and desorption phenomena of hydrogen and oxygen do not overlap. For all of these reasons, this metal is particularly suitable for studying the behavior of this type of sensor in porous media. Its high stability and electrocatalytic character make it especially advantageous for understanding the performance of voltammetric sensors embedded in porous materials.

At present, gold has been widely used in the fabrication of sensors for both analytical chemistry^58,59^ and the food industry.^60^ In most cases, these are electrochemical sensors.^59^ In the case of concrete, gold has been applied in the development of fiber-optic,^20,34,61^ potentiometric,^20^ and amperometric sensors.^20,62,63^

## 2 Experimental Section

### 2.1 Gold Sensor Electrode

For the fabrication of the gold electrode, a polycrystalline Au wire (99% purity) with a diameter of 1 mm was used. Electrical connection was made by using a multistrand wire insulated with Teflon. The junction between the wire and the metal was protected with heat-shrink tubing and epoxy resin (Figure 1). The effective surface area of the electrodes used was 0.07 ± 0.02 cm^2^.

**Figure 1:**
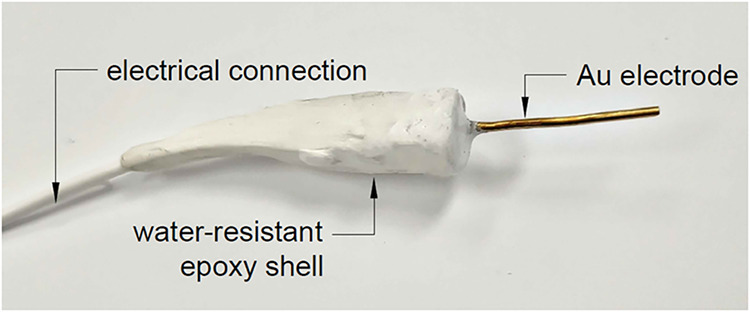
Photograph of the gold (Au) electrode.

### 2.2 Electrochemical Techniques

Cyclic voltammetry (CV) was performed using an Autolab PGSTAT10 potentiostat, and data were acquired with Nova 1.11 software.

A three-electrode cell configuration was employed, where the gold electrode served as the working electrode (WE), stainless steel plates (SS) were used as the counter electrode (CE), and a saturated calomel electrode (SCE) was used as the reference electrode. The surface area of the CE was maintained at least 40 times greater than that of the WE to prevent reactions at the counter electrode from influencing the recorded response.^48^ The scan rate was set to 20 mV·s^–1^.

To avoid altering the local O_2_ availability near the sensor, which may occur due to water oxidation, each potential sweep was initiated at 0 V vs SCE and proceeded in the cathodic direction to −1.2 V vs SCE.^5^ Potentials more negative than −1.2 V were not explored since water reduction occurs beyond this potential under alkaline conditions, leading to the evolution of H_2_ and OH^–^ and promoting H_2_O_2_ formation. This could interfere with the interpretation of the sensor response, as hydrogen peroxide undergoes a catalytic reaction on the Au surface.^5,64^

Electrochemical impedance spectroscopy (EIS) was performed in the frequency range from 100 to 1 kHz, using a 10 mV AC perturbation superimposed on a DC bias of 0 V vs SCE, in order to determine *R*_u_.^65^ This value was used to correct for the ohmic drop in the voltammograms. *R*_u_ was calculated using the simplified Randles equivalent circuit (*R*_s_–(*R*_p_/*C*_dl_)).

Consistent with published works,^65–67^ the notation used in the voltammetric plots designates the applied potential as Δ*E*_RW_ when the ohmic drop was uncompensated and as Δ*E*_WE_ when the ohmic drop was compensated. The current response in the CV experiments is denoted as *i*, while *j* represents the current density, i.e., the current normalized by the WE surface area.

### 2.3 Experimental Section

To evaluate the effectiveness of the voltammetric sensor response in characterizing the composition of the pore solution in porous materials, the response of the embedded voltammetric sensor was compared across concrete specimens subjected to different conditions (water-saturated state, chloride contamination within the pore network, and carbonated concrete). These responses were compared with those obtained from the same sensor immersed in solutions simulating the corresponding pore-solution conditions of concrete.

By comparing the voltammograms recorded in solution with those obtained in concrete, similarities and differences between both environments were identified, allowing assessment of the sensor’s capability to characterize a porous medium containing capillary moisture.

#### 2.3.1 Studies in Solution

The experiments in solution were performed using the same three-electrode configuration described in the Electrochemical Techniques section, with the gold electrode as the working electrode (WE), stainless steel plates as the counter electrode (CE), and a saturated calomel electrode (SCE) as the reference. The electrodes were placed in a laboratory cell of approximately 100 mL (Figure 2). No stirring or rotation was applied in order to reproduce conditions as similar as possible to those expected when the sensor is embedded in concrete. Argon bubbling was carried out for 30 min when oxygen removal was required; otherwise, the cell was left open to air. The geometry and relative position of the electrodes were kept constant in all experiments, so that variations in *R*_u_ would mainly reflect electrolyte composition rather than geometric effects.

- Baseline response in alkaline medium. The voltammetric response of the gold sensor was first examined with the electrode immersed in a 0.1 M KOH solution, which simulates the pH of the concrete pore solution under normal conditions (pH ≈ 12.5). Measurements were carried out both under aerated conditions and after deoxygenation.
- Simulated pore-solution conditions. Different solutions were then prepared to simulate various conditions representative of the concrete pore solution. All measurements in these solutions were conducted under synthetic air:

(1) M KOH (pH = 12.5): simulating noncarbonated concrete.^68^
(2) M NaHCO_3_ (pH = 8.15): simulating carbonated concrete.^68^
(3) M NaOH (pH = 12.7) + 0.5 M NaCl (pH = 12.7): prepared to evaluate the influence of chloride ions on the obtained results.

**Figure 2:**
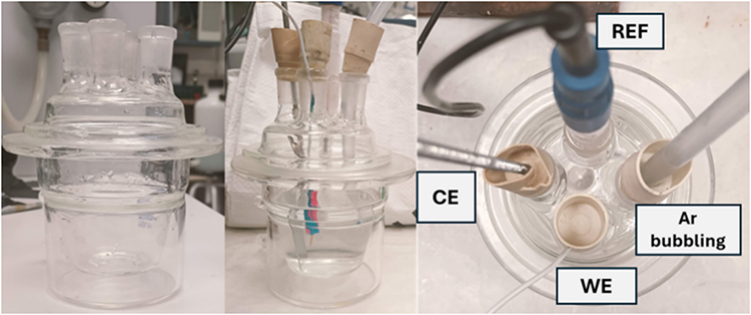
Photographs of the electrochemical cell used for measurements in solution. The three-electrode configuration is shown, including the gold working electrode (WE), the counter electrode (CE), and the saturated calomel reference electrode (REF). Argon bubbling was used to control the oxygen availability when required.

The results were compared with literature data to better understand the interaction of the Au sensor with the various ions analyzed.

#### 2.3.2 Studies in Concrete

Concrete specimens measuring 4 × 4 × 16 cm^3^ were fabricated by using the mix design specified in Table 1. A high water-to-cement ratio (w/c) concrete was employed to achieve medium-to-low porosity, thereby facilitating controlled modifications of the material’s condition in the hardened state.

**Table 1:** Concrete Specifications

Materials	kg/m^3^_of concrete_
Cement I 42.5 R-SR5	315
Water	189
Superplastifier	2.2
Silica sand	1212
Gravel	653
w/c	0.6
Accessible porosity for water (UNE 83980:2014).	19.19%

Porosity was evaluated according to the standardized water absorption test UNE 83980:2014. For this purpose, three cylindrical specimens (10 cm in diameter and 5 cm in height) were prepared from the same concrete mixture. The results were classified following the durability criteria proposed by the AFGC and the exposure classes defined in Eurocode 2.^69^ The coefficient of variation of the measurements was 5%, indicating good repeatability of the test.

Each specimen contained an embedded gold electrode (WE) and a stainless steel plate serving as the counter electrode (CE). The arrangement of the electrodes within the specimens is shown in Figure 3. During the CV experiments, the reference electrode (SCE) was positioned as illustrated in Figure 3.

**Figure 3:**
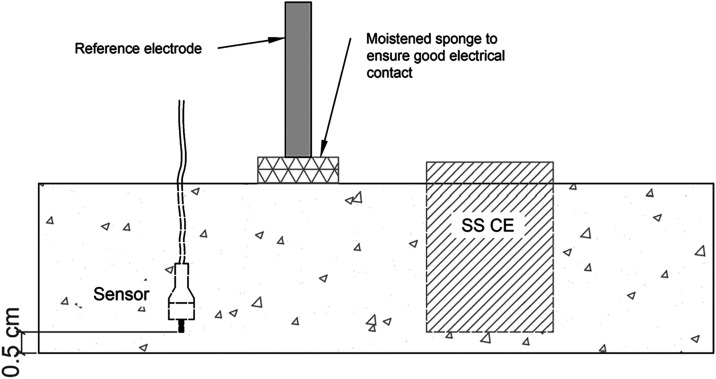
Schematic representation of the concrete specimens used in the study, illustrating the spatial arrangement of the embedded gold working electrode (WE), the stainless steel counter electrode (CE), and the position of the reference electrode (SCE) during cyclic voltammetry measurements.

Three specimens were prepared for each concrete conditioning type defined in Table 2. In total, 12 specimens were fabricated, each containing an embedded gold electrode.

**Table 2:** Specimen Conditioning Conditions

Conditioning	Conditions	Acronym
After removal from the curing chamber, specimens were exposed to laboratory ambient conditions (*T* ≈ 25 °C; RH ≈ 60%) until the weight variation between two consecutive days was less than 2%.	This condition corresponds to the normal pH of the concrete pore solution (pH ≈ 12.5) under ambient humidity.	AH
After curing, specimens were immersed in a saturated Ca(OH)_2_ solution to prevent OH^–^ leaching. Immersion was continued until the weight variation between two consecutive days was less than 2%.	This condition corresponds to the normal pore-solution pH (pH ≈ 12.5) under full water saturation.	WS
After curing, specimens were immersed in a 0.5 M NaCl solution. Measurements were performed until stabilization of the peaks observed in the voltammogram was achieved.	This condition simulates the presence of chlorides in the capillary network of concrete (pH ≈ 12.5).	CP
Specimens were exposed to a 3.5% CO_2_ atmosphere for sufficient time to achieve complete carbonation.	This condition corresponds to carbonated concrete with a pore-solution pH below 8.5.	CC

Before being conditioned, all specimens were cured in a controlled chamber for 28 days at approximately 22 °C and 99% relative humidity (RH).

### 2.4 Data Analysis

First, the voltammograms recorded in solution and in concrete were qualitatively compared to analyze the electrochemical response patterns and identify similarities and differences in the sensor behavior between aqueous and porous environments.

Subsequently, the dependence of characteristic parameters, current densities, peak potentials, and peak separations on the scan rate and medium composition was evaluated to determine the prevailing control mechanisms (diffusion, kinetic, or mixed).

In addition, the onset potential was determined by a linear extrapolation. The linear portion of the rising current was extrapolated back to the extrapolated baseline current, and the corresponding intercept was taken as the onset potential.

Finally, PCA was applied to discriminate among different concrete conditions (carbonated, chloride-contaminated, and reference) and to assess the reproducibility and discrimination capability of the embedded sensor response.

The data-processing protocol was implemented using the software Solo 9.0 (2022) (Eigenvector Research, Inc., Manson, WA, USA; available at http://www.eigenvector.com).

As described in the previous section, the concrete specimens were grouped into four different conditioning states. For the PCA, each individual cyclic voltammogram was treated as one observation. Two repeated measurements were performed on each specimen, resulting in six observations per conditioning state and a total of 24 observations. The resulting data matrix is summarized in Table 3, where each row corresponds to one voltammetric measurement. The “Condition” column indicates the concrete state, and the remaining columns contain the current density values sampled at each potential within the selected scan window.

**Table 3:** Data Matrix used for Principal Component Analysis (PCA)^a^

		*j*(Δ*E*_RW_)/μA·cm^–2^										
Samples	Condition	*j*(0.000 V)	···	*j*(−0.005 V)	···	*j*(−1.500 V)	···	*j*(0.600 V)	···	*j*(0.010 V)	···	*j*(0.000 V)
S_1_	AH	–0.56	···	–0.35	···	–145.57	···	4.58	···	–2.18	···	–2.44
···	···	···	···	···	···	···	···	···	···	···	···	···
S_7_	WS	1.24	···	0.90	···	–671.34	···	162.36	···	–131.87	···	–135.05
···	···	···	···	···	···	···	···	···	···	···	···	···
S_13_	CP	0.11	···	0.11	···	–867.83	···	244.83	···	–133.39	···	–119.85
···	···	···	···	···	···	···	···	···	···	···	···	···
S_19_	CC	–0.03	···	0.00	···	–1.19	···	0.11	···	–0.13	···	–0.06
···	···	···	···	···	···	···	...	···	···	···	...	...
S_24_	···	···	···	···	···	···	...	···	···	···	...	...

a AH: air-saturated; WS: water-saturated; CP: carbonated; CC: chloride-contaminated. Current densities were sampled every 5 mV within the selected potential window.

## 3 Results and Discussion

### 3.1 Analysis of Voltammogram Morphology

#### 3.1.1 Study in Base Solution (0.1 M KOH)

Figure 4 shows a typical voltammogram of Au in an alkaline medium.^5,54,55,70^ Between the water oxidation and reduction curves, non-Faradaic processes of electrical double-layer charging and discharging occur, accompanied by Faradaic processes described in the literature, associated with electrolytic reactions of various ions: the formation of Au­(OH), oxygen adsorption (Au–O), and the initial formation of Au­(II) and Au­(III) oxides.^5,54,55^ According to previous studies, peaks A and B are attributed to the formation of adsorbed Au–OH species, resulting from partial oxidation of the gold surface prior to the growth of thicker oxides observed at higher potentials.^71–74^ Conversely, peaks a and b are related to the desorption of OH^–^ ions generated during the reduction of oxides and species formed in processes A and B.^5,70,75^

**Figure 4:**
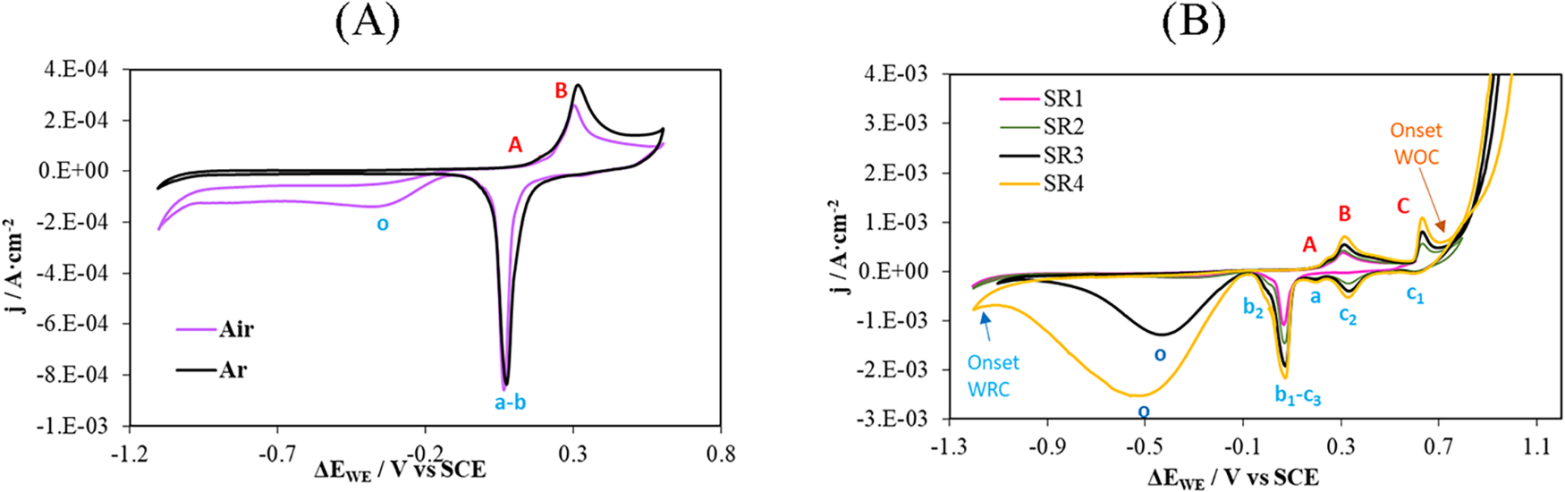
Voltammograms obtained with a gold (Au) electrode in a 0.1 M KOH solution. The reference electrode used was SCE. (A) Voltammograms recorded within the sweep range from 0.6 V to −1.1 V under deoxygenated atmosphere (Ar, black line) and air-saturated atmosphere (Air, purple line). (B) Voltammograms recorded at different sweep ranges (SR). SR1:0.6 V to −1.2 V; SR2:0.8 V to −1.2 V; SR3:1.0 V to −1.2 V; SR4:1.1 V to −1.2 V. WRC: water reduction curve. WOC: water oxidation curve.

Starting from peak B, oxygen adsorption on the Au surface begins. Despite the decrease in current density beyond peak C (Figure 4B), the literature reports that during the water oxidation curve, and from a potential of approximately 1 V vs SCE in alkaline media, the passive layer of Au_2_O_3_ begins to form.^54,55,71–74,76^ In all cases, potential sweeps were applied such that the maximum anodic potential did not exceed the onset of passive layer formation (with a maximum of +1.1 V vs SCE).

The objective of this study was not to investigate the formation of Au oxides but rather to evaluate the sensor’s response under various conditions that could affect its performance. This approach enabled the identification of behavior patterns that contribute to understanding sensor behavior in porous materials, such as concrete.

Figure 4A displays the voltammograms obtained using the Au sensor when the sample transitions from a deoxygenated atmosphere (black line) to an air-saturated atmosphere (purple line). The figure reveals a significant increase in current density in the region where the o reduction peak appears. Considering the conditions of both experiments, this increase can only be attributed to the reduction of O_2_, since N_2_ is an inert gas. This observation is consistent with findings reported by Srejić et al.^5^

Regarding the reduction of dissolved oxygen on gold, two main pathways have been proposed, both involving adsorbed intermediates and the formation of peroxide species. Following the mechanism described by Damjanović et al.,^77^ oxygen can be reduced either through proton-assisted superoxide formation or via direct superoxide generation. In the present work, the appearance of cathodic features in aerated alkaline solution is consistent with a surface-controlled oxygen reduction process on Au, in which adsorbed O_2_^–^ and OOH^–^ species may participate. This is also coherent with the disappearance or attenuation of these peaks under Ar purging.

#### 3.1.2 Study in Concrete under Standard Conditions

The ohmic drop is greater in concrete than in aqueous solution due to the lower electrical conductivity of the porous medium. In this case, the uncompensated resistance (*R*_u_) measured in solution was approximately 3% of the *R*_u_ obtained in concrete. In an aqueous solution, ions are free to move, which facilitates efficient ionic conduction and results in lower electrolyte resistance. In contrast, in concrete, the pores are partially filled with a pore solution that contains a lower concentration of mobile ions. Additionally, the tortuosity of the porous medium further increases the effective electrical resistance between the working and reference electrodes.

As a consequence, the potential drop due to electrolyte resistance (*iR* drop) is more pronounced in concrete than in solution. This means that the effective potential applied to the working electrode (Δ*E*_WE_(*t*)) is lower than the set point potential signal (Δ*E*_RW_(*t*)) in the case of concrete. This effect is especially significant in the potential regions where the current density (*j*) is high, namely, in the zone corresponding to O_2_ reduction and the onset of the water reduction curve, as well as under ambient concrete conditions, where the ohmic drop is even more substantial (Figure 5).

**Figure 5:**
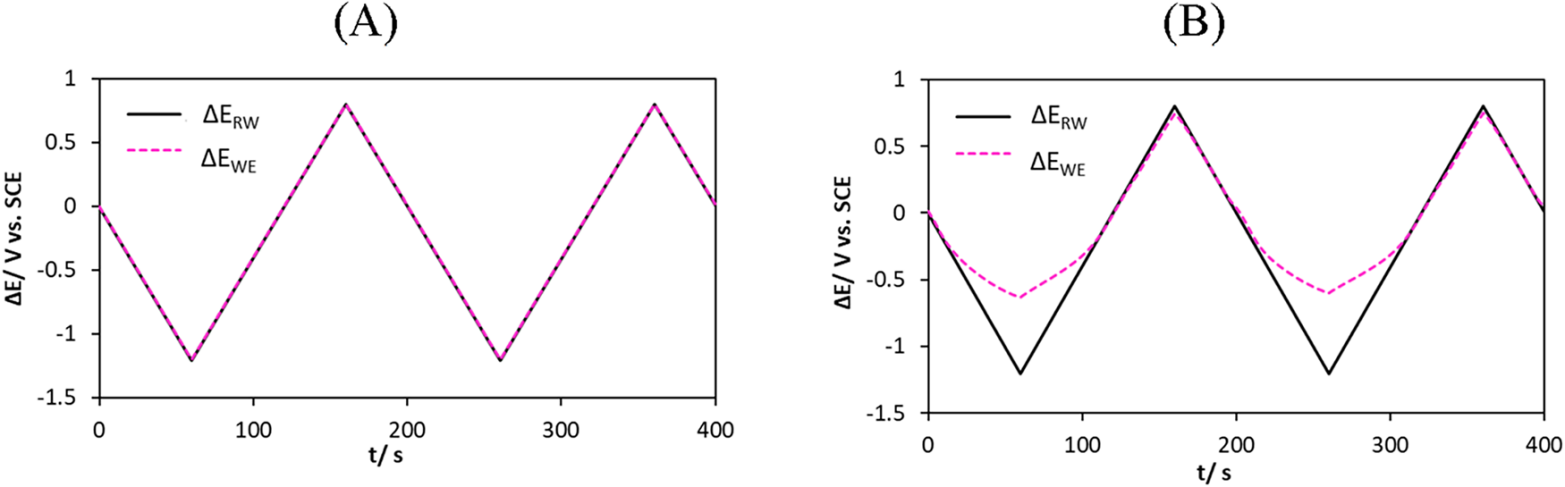
Comparison between the applied potential signal measured between the working and reference electrodes (Δ*E*_RW_) and the corrected potential after ohmic drop compensation (Δ*E*_WE_). (A) Electrode immersed in aqueous solution. (B) Electrode embedded in concrete under atmospheric conditions (AH).

Figure 6 shows the voltammograms obtained for the sensor embedded in concrete under typical laboratory working conditions. Two moisture conditions within the porous network were studied: water saturation (Figure 6A, WS) and ambient conditions (Figure 6B, AH, ambient conditions: *T* = 25 ± 3 °C, RH ≈ 60%). The four potential sweep ranges defined in the dissolution study (Figure 4B) were applied.

**Figure 6:**
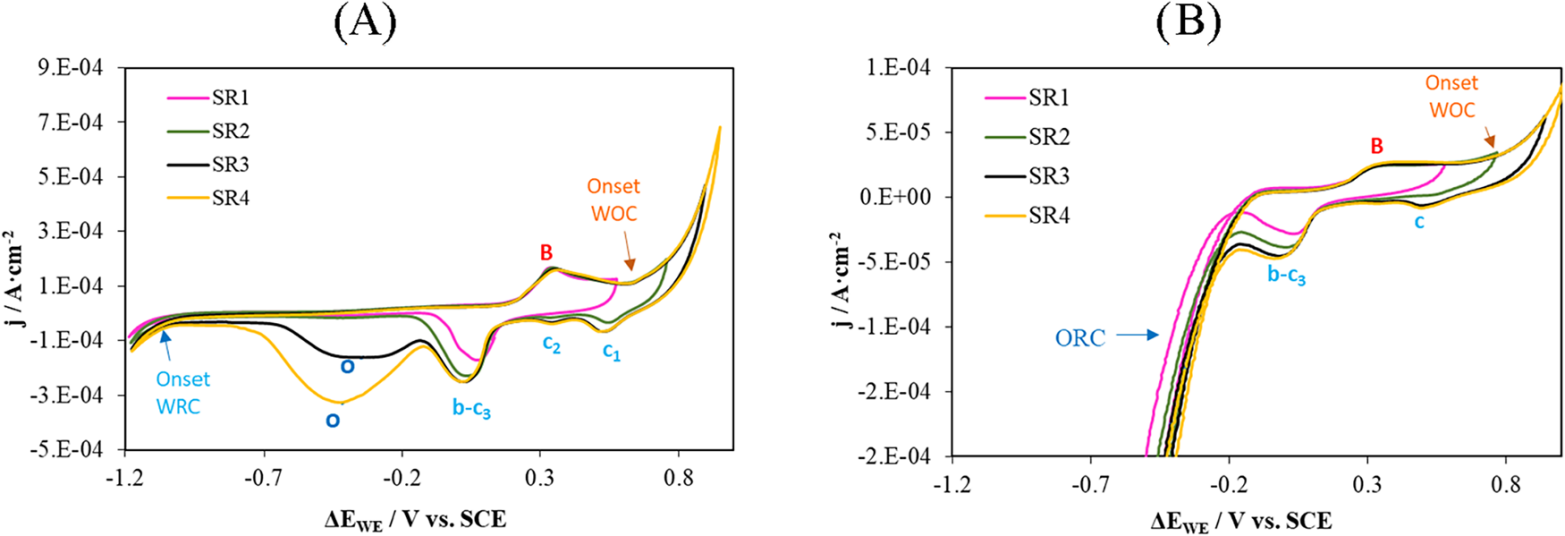
Voltammograms obtained with the Au sensor embedded in concrete. (A) Under water saturation conditions (WS). (B) Under ambient conditions (AH). SR: Sweep ranges. SR1:0.6 V to −1.2 V; SR2:0.8 V to −1.2 V; SR3:1.0 V to −1.2 V; SR4:1.1 V to −1.2 V. WRC: Water reduction curve. WOC: Water oxidation curve. ORC: Oxygen reduction curve.

When the voltammograms obtained in concrete are compared with those recorded in solution (Figure 4), peaks B and b–c_3_ appear at similar potentials under saturated concrete conditions. However, under ambient conditions, the b–c_3_ peak shifts to more negative potentials. Peak A is not observed in concrete, nor is peak C, which is overlapped by the water oxidation curve.

The variation in current density and corrected potential (Δ*E*_WE_) for peak B remains consistent across sweep ranges, whereas peak b–c_3_ is strongly affected by the sweep range, as was also observed in solution.

Another important observation is that in concrete, the oxygen reduction reaction on the Au sensor surface remains favored (peak o).

#### 3.1.3 Effect of Carbonation

When concrete undergoes carbonation, carbon dioxide (CO_2_) primarily reacts with calcium hydroxide (Ca­(OH)_2_) present in the pore solution, leading to the formation of calcium carbonate (CaCO_3_). This compound has low solubility and can result in a reduction of the pore size or even pore blockage, accompanied by a decrease in pH due to the neutralization of hydroxide ions (OH^–^) in the pore solution.

To assess whether the electrochemical changes observed in solution are also replicated within the concrete matrix, Figure 7A presents a comparison of voltammograms obtained in synthetic pore solutions simulating noncarbonated (pH ≈ 12.5) and carbonated (pH ≈ 8.35) concrete. Figure 7B shows the corresponding voltammograms recorded by sensors embedded in concrete under the same conditions.

**Figure 7:**
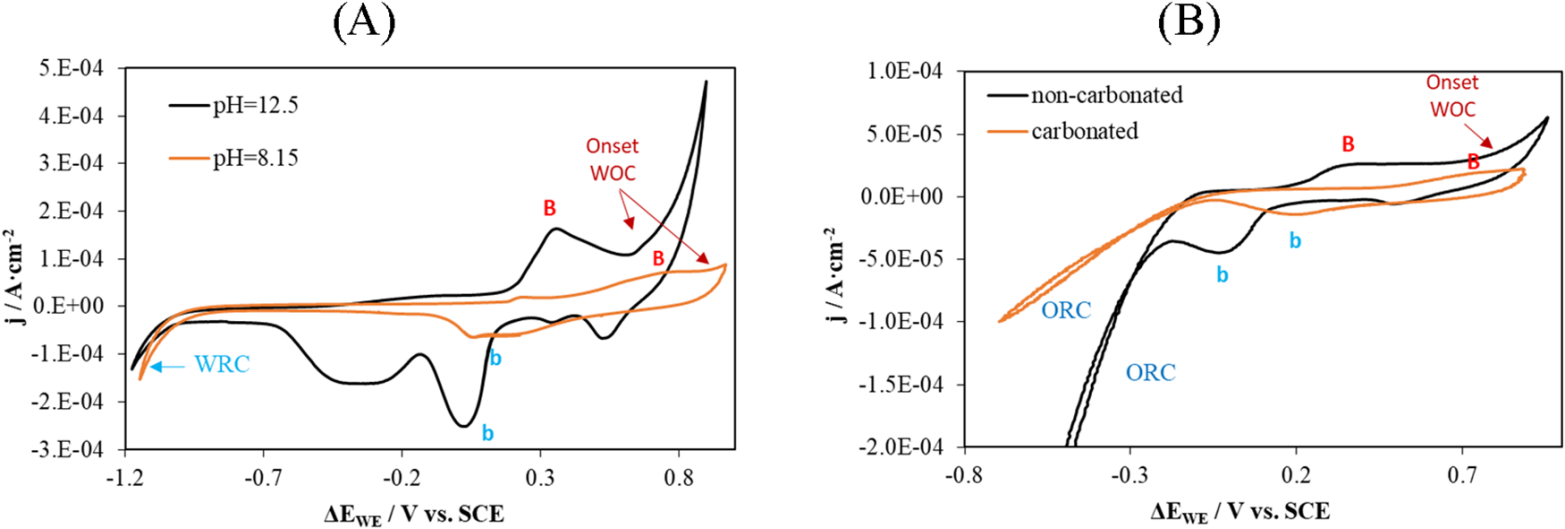
Cyclic voltammetry results obtained with a gold working electrode at different pH values. The reference electrode used was SCE. (A) Measurements in solution: pH = 12.5 (0.1 M KOH, black line) and pH = 8.15 (0.1 M NaHCO_3_, orange line), under aerated conditions. (B) Measurements in noncarbonated concrete (black line) and carbonated concrete (orange line). WRC: water reduction curve; WOC: water oxidation curve; ORC: oxygen reduction curve.

The degree of carbonation in the specimens was confirmed via a colorimetric test using phenolphthalene (Figure 8). After cross-sectioning the specimens and applying the indicator solution, carbonated regions (pH < 9) remained colorless, whereas noncarbonated regions (pH > 11) turned fuchsia, confirming complete carbonation.

**Figure 8:**
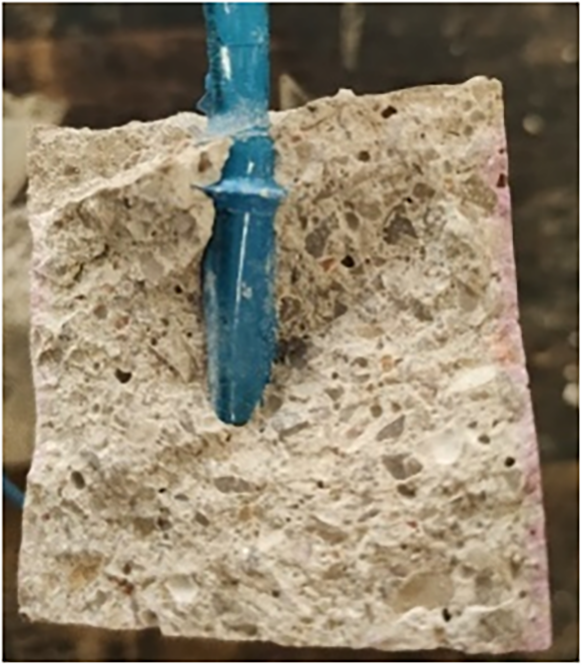
Photograph of one of the specimens sprayed with phenolphthalein.

A comparison between measurements in solution and in concrete reveals that as pH decreases, the voltammograms in both media exhibit similar shifts in redox peak positions toward more positive potentials in both the anodic and cathodic branches, as well as in the onset potentials of the water oxidation and reduction curves. This behavior is consistent with the findings reported by Angerstein-Kozlowska et al.^55^ and Nicol.^70^

In the case of the sensor embedded in concrete, the current intensity is lower, with broader and less pronounced peaks, as a consequence of the increased ionic resistance of the material. This effect is more pronounced in carbonated concrete, where the uncompensated resistance (*R*_u_) reaches values on the order of 10^5^ Ω, compared to approximately 10^4^ Ω in noncarbonated concrete. The increase in *R*_u_ limits both the charge transfer and diffusion processes, resulting in an overall attenuation of the redox peaks.

#### 3.1.4 Effect of Chloride Presence

The ionic composition of the electrolyte in which the gold electrode is immersed has a strong influence on its electrochemical response.^55^ In the pore solution of concrete, various species may coexist, primarily hydroxides and carbonates, and under aggressive environmental conditions, chlorides may also be present. Therefore, the influence of Cl^–^ ions on the sensor response was investigated.

Figure 9A shows the study conducted in solution regarding the effect of increasing the Cl^–^ concentration on the response of the Au sensor. The introduction of chlorides leads to the appearance of a new pair of peaks, labeled E–e, with significantly higher current density compared to the others. Additionally, peak B shifts toward more positive potentials and its intensity decreases markedly, resulting in the formation of peak B′. Moreover, peak b, associated with the desorption of OH^–^ and the O_2_ species, nearly disappears in the presence of chlorides. This behavior is consistent with the findings of Angerstein-Kozlowska et al.,^55^ who reported that the oxides formed on Au are strongly affected by the adsorption of anions on its surface.

**Figure 9:**
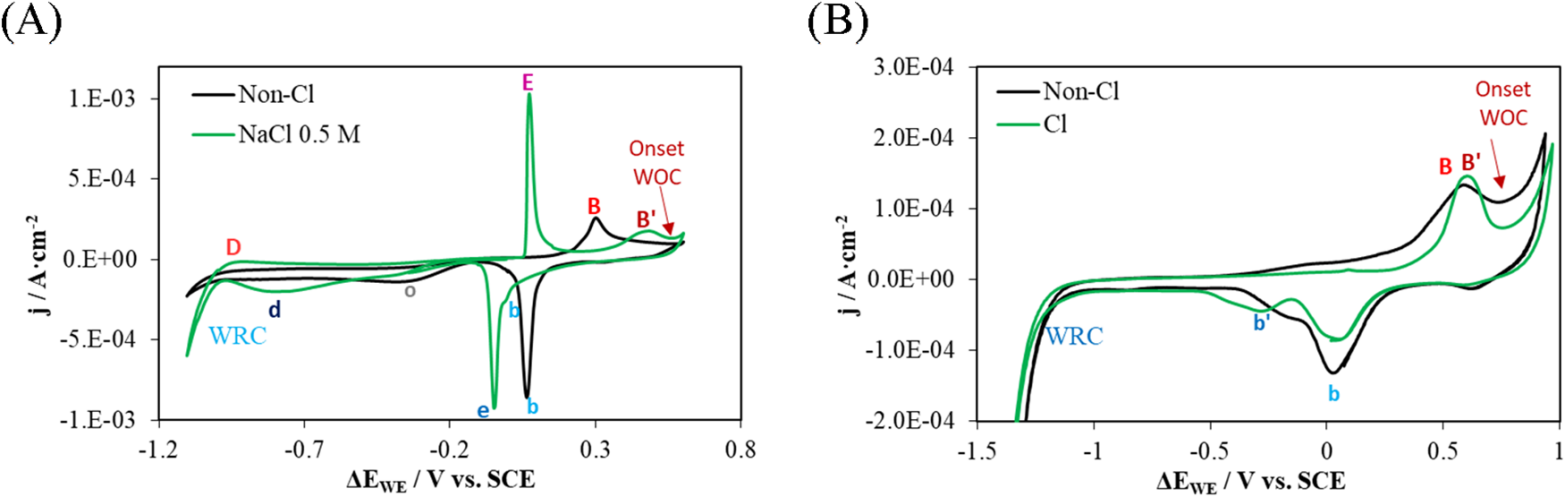
Cyclic voltammetry results obtained with a gold working electrode at different chloride concentrations. The reference electrode used was SCE. (A) Measurements in solution: 0.1 M KOH (Non-Cl; black line) and 0.1 M KOH + 0.5 M NaCl (0.5 M NaCl; green line) under aerated conditions. (B) Measurements in concrete without chlorides (Non-Cl; black line) and with chlorides (Cl; green line). WRC: water reduction curve; WOC: water oxidation curve.

Several studies have addressed the interaction between gold and chlorides, given that chlorination is one of the methods used for gold dissolution and extraction.^78–80^ In particular, Aldous et al.^81^ associated the appearance of additional voltammetric peaks with the adsorption reaction and formation of gold-chloride species. Based on these results, it can be stated that peak E is related to the concentration of chlorides present in the solution and that the adsorption of OH^–^ and O_2_ (peak B) is modified by the presence of this anion, to the extent that peak b, associated with the desorption of OH^–^ and O_2_, nearly disappears.

To evaluate this effect, in concrete, the ionic concentration of the specimens was increased by saturating them with a 0.5 M NaCl solution. During the exposure period, chlorides penetrated the concrete matrix and reached the sensor zone. Figure 10 shows an example of a specimen exposed to a chloride-rich environment. The presence of free chlorides around the sensor was confirmed by spraying AgNO_3_ onto the cross-sectioned surface: a whitish coloration indicates the formation of AgCl and, therefore, the presence of chlorides, whereas darkening reveals their absence. In the specimens studied, the whitish coloration confirmed the complete chloride penetration.

**Figure 10:**
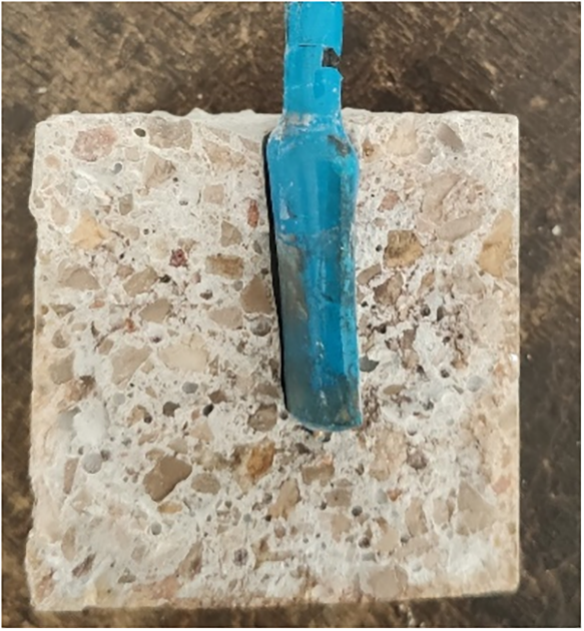
Photograph of one of the specimens sprayed with AgNO_3_. The entire section exhibits a homogeneous, whitish appearance associated with total chloride contamination.

Based on the results obtained in solution, an increase in current density and the appearance of new peaks associated with gold-chloride species would be expected (Figure 9A). However, in embedded concrete, no new peaks or appreciable increase in current density were observed (Figure 9B). Although peak B becomes sharper and the morphology of the voltammogram changes, the presence of chlorides leads to the formation of new products on the electrode surface. Combined with the inhibition of diffusion phenomena due to the compactness of concrete, compared to the free diffusion environment of a solution, this causes the electrode response to chlorides to differ from that observed in aqueous media. A new peak, b′, is observed.

### 3.2 Comparison of Electrochemical Behavior

To compare the phenomena governing the reaction in both media, the redox peak pair B-b, identified in both environments, was selected. The influence of the medium and the scan rate on the voltammetric response of the system was studied by recording cyclic voltammograms in aqueous 0.1 M KOH solution and in a concrete specimen, with varying scan rate (Figure 11A,B, respectively).

**Figure 11:**
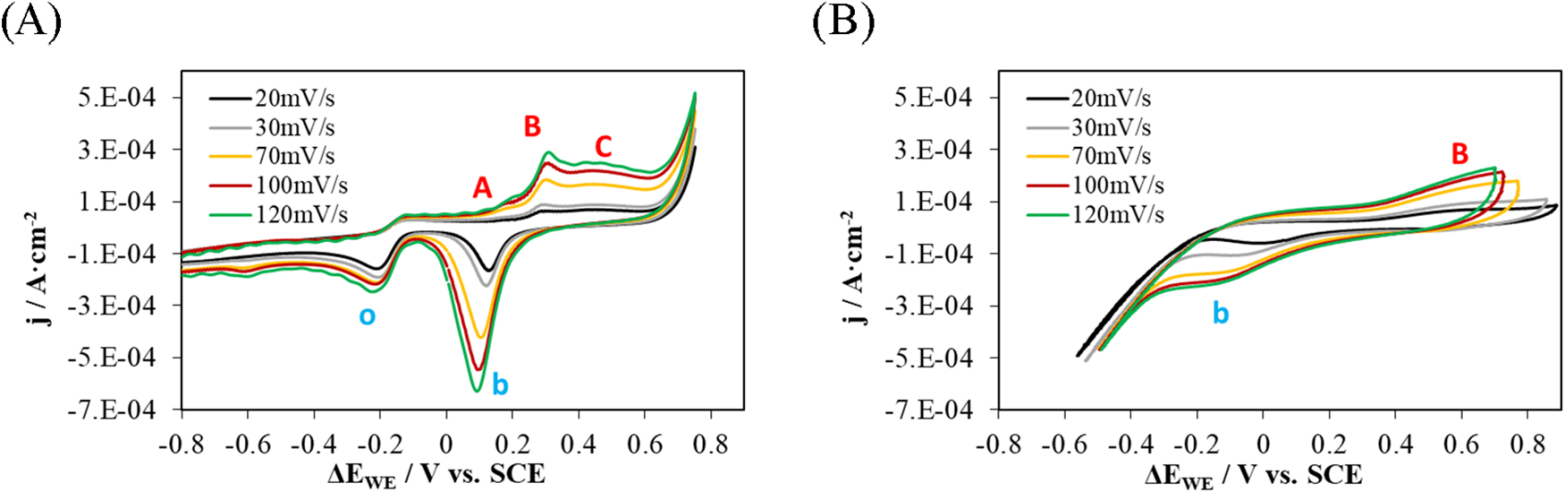
Voltammograms obtained with the Au sensor at different potential scan rates (20, 30, 70, 100, and 120 mV·s^–1^). (A) In a 0.1 M KOH solution under aerated conditions. (B) Sensor embedded in concrete.

In both cases, the voltammograms shown correspond to *iR*-corrected data. Although the same potential window was applied experimentally, the much higher ohmic resistance of concrete causes the *iR* correction to produce a more noticeable apparent change in the potential window as the scan rate increases.

The analysis of the peak current dependence on the scan rate was interpreted using the Randles–Sevcik model to determine the dominant transport regime: either diffusion-controlled or adsorption-controlled.

Additionally, the variation of the peak potential with the logarithm of the scan rate was analyzed using the Laviron model, which allows for the evaluation of the contribution of charge transfer kinetics. The results obtained in concrete were compared to those from the solution.

Regarding the results obtained in solution, peak B shows a very strong linear dependence both with the scan rate (*v*) (*R* ^2^ = 1.00) (Figure 12A) and with its square root (√*v*) (*R* ^2^ = 0.99)­(Figure 12C), as well as a slope of 0.83 in the log |*j*| vs log *v* plot (Figure 12E). Peak b exhibits similar behavior, with a good fit to both the scan rate and its square root (*R* ^2^ ≈ 0.99) (Figure 12A y C), and a slope of 0.74 in the log |*j*| versus log *v* plot. The strong correlation with *v* and √*v*, along with slope values between 0.5 and 1.0, indicates a mixed process, diffusion-controlled with some surface or kinetic contribution.

**Figure 12:**
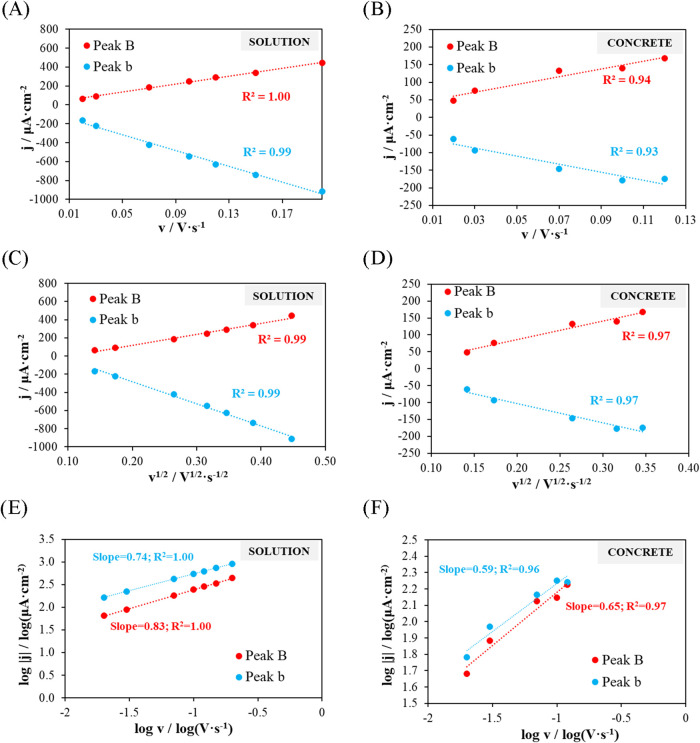
Influence of scan rate on peak current density. (A, B) Current density versus scan rate for the solution and concrete, respectively. (C, D) Current density versus the square root of the scan rate for the solution and concrete, respectively. (E, F) Logarithm of the absolute value of the current density versus the logarithm of the scan rate for the solution and concrete, respectively.

In the plot of peak potential versus log v, peak B does not show a clear dependence (*R* ^2^ ≈ 0.66), whereas peak b does (*R* ^2^ ≈ 0.99) (Figure 13A). Regarding the kinetic analysis using the Laviron model and assuming *T* = 25 °C, the calculated values are α*n* = 2.96 for peak B and α*n* = 1.18 for peak b (Table 4), suggesting that the reduction/desorption reaction exhibits a more significant kinetic component.

**Figure 13:**
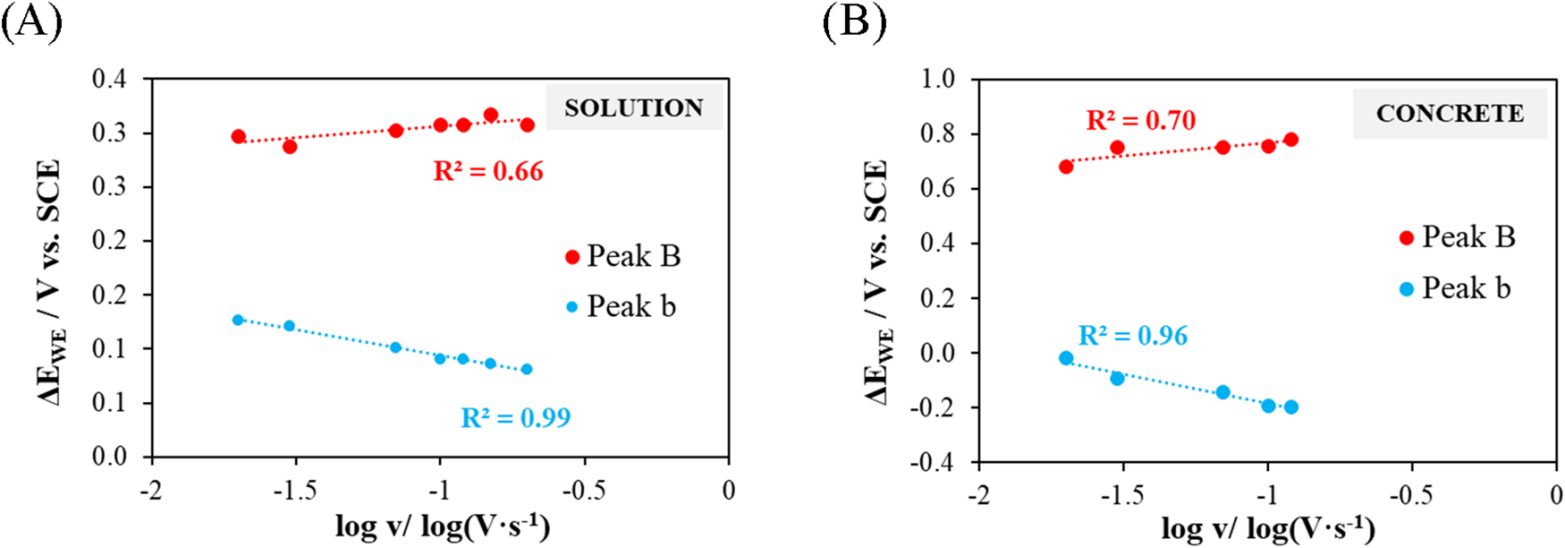
Influence of scan rate on peak potential (*iR* drop compensated). (A) and (B) Peak potential versus the logarithm of the scan rate for the solution and concrete, respectively.

**Table 4:** α*n* Values Obtained through the Analysis of Δ*E*_p_ vs log*v*

Media	Peak	|Slope| (V/dec)	α*n*
Dissolution	B	0.02	2.96
	b	0.05	1.18
Concrete	B	0.09	0.66
	b	0.22	0.27

The potential separation Δ*E*_p_ = 0.19 ± 0.02 V and the current ratio *i*_B_/*i*_b_ = 0.43 ± 0.03 indicate a quasi-reversible process with fast but asymmetric electron transfer, where reduction is more favored than oxidation.

Experiments conducted at different KOH concentrations show that the peak current density varies only slightly with concentration (*R* ^2^ = 0.41 for peak B and 0.54 for peak b; Figure 14A) and that the peak potential does not exhibit a clear relationship either (*R* ^2^ ≤ 0.8, Figure 14B). This indicates that beyond 0.1 M, the process is not limited by the availability of OH^–^ ions but rather by transport and adsorption factors at the surface of the gold electrode.

**Figure 14:**
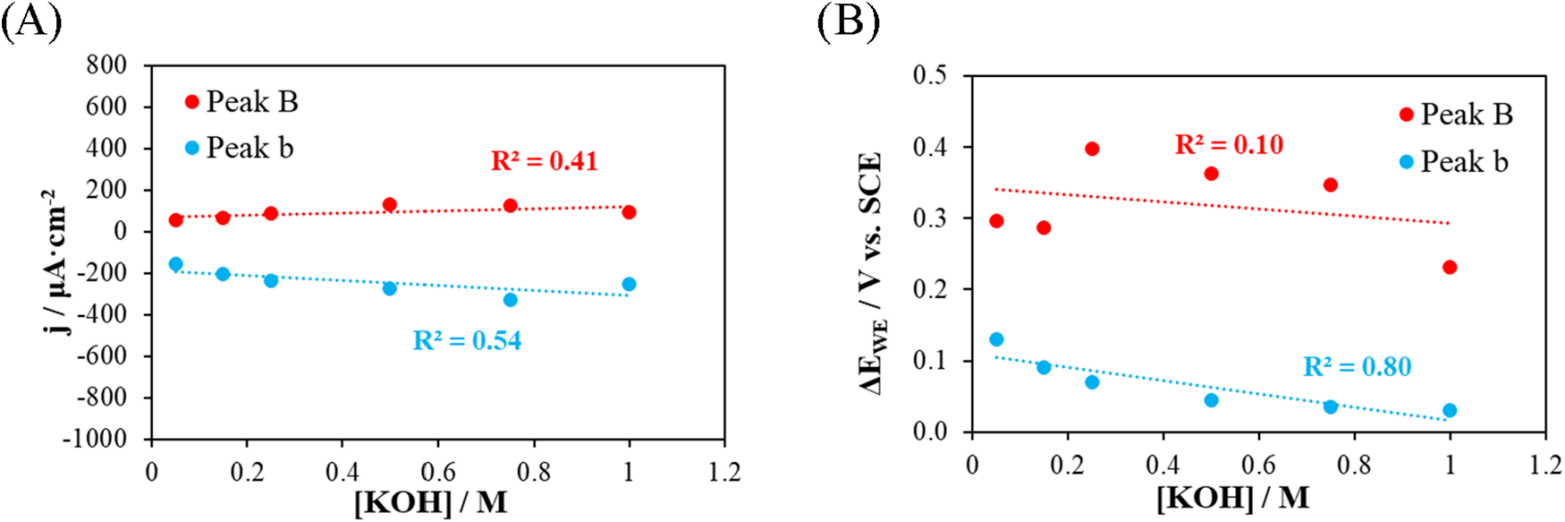
Influence of analyte concentration on the electrochemical response of the voltammetric sensor. (A) Peak current density as a function of analyte concentration. (B) Peak potential as a function of analyte concentration.

As for the results obtained in concrete, both peaks show a good linear relationship with the scan rate (*v*), similar to the behavior observed in solution (*R* ^2^ > 0.93; Figure 11B) and an even slightly better correlation with its square root (√*v*) (*R* ^2^ ≈ 0.97; Figure 11D). The slopes of log|j| versus log *v* are 0.65 for peak B and 0.59 for peak b, slightly lower than those obtained in solution. This indicates a predominantly diffusion-controlled process, although with some additional kinetic limitations, consistent with transport through the tortuous porous network of concrete.

In the plot of peak potential versus log *v*, both peaks exhibit a similar degree of linear correlation as in solution (Figure 13), with *R* ^2^ = 0.70 for peak B and 0.96 for peak b, reflecting a greater kinetic control in the reduction/desorption process, as also observed in solution.

Based on the slopes obtained and considering the same conditions as in solution, the αn values were calculated using the Laviron model (Table 4), resulting in 0.66 for peak B and 0.27 for peak b, both lower than those obtained in solution. These results confirm that, in concrete, the reaction is more limited by charge transfer kinetics than in solution, particularly in the cathodic branch.

The corrected peak separation Δ*E*_p_ = 0.87 ± 0.11 V (*iR*-compensated) is significantly higher than that in solution, further indicating slower kinetics. Meanwhile, the current ratio *i*_B_/*i*_b_ = 0.85 ± 0.08 suggests greater symmetry between the oxidation and reduction currents. Additionally, the peaks are broader and less defined than those in solution (Figure 11A,B), reinforcing the notion of a slower and strongly polarized process, influenced by the high ionic resistance (*R*_u_ ≈ 20 kΩ) and restricted diffusion within the porous structure of the material.

In both media, the B-b redox pair corresponds to the same OH^–^ adsorption/desorption process on the gold surface; however, the surrounding environment determines the nature of the controlling mechanism.

In solution, the process is quasi-reversible and mixed diffusion-kinetic with fast electron transfer and low polarization (Δ*E*_p_ = 0.19 V, α*n* ≈ 1–3).

In concrete, the process becomes predominantly diffusion-controlled with strong kinetic limitations, as reflected by the large peak separation (Δ*E*_p_ = 0.87 V) and low αn values, ranging from 0.2 to 0.7.

The peak morphology, sharper in solution and broader in concrete, and the difference in ionic resistance (*R*_u solution_ ≈ 10^2^ Ω vs *R*_u concrete_ ≈ 2 × 10^4^ Ω) confirm that the limitations in concrete are mainly due to ionic transport and the resistivity of the medium, rather than the intrinsic kinetics of the gold electrode.

### 3.3 PCA Analysis

Figure 15 shows the PCA score plot, which illustrates the distribution of samples in the plane defined by the first two principal components for all concretes under the four analyzed conditions. The *X*-axis corresponds to PC1, which accounts for 45.48% of the total variance, while the *Y*-axis represents PC2, explaining 36.71%. Together, both components capture 82.19% of the total data variance. This value exceeds the 70% threshold typically considered acceptable for a reliable representation of the data set.^42,43,46,82,83^ Therefore, the PCA model adequately describes the variability among the samples.

**Figure 15:**
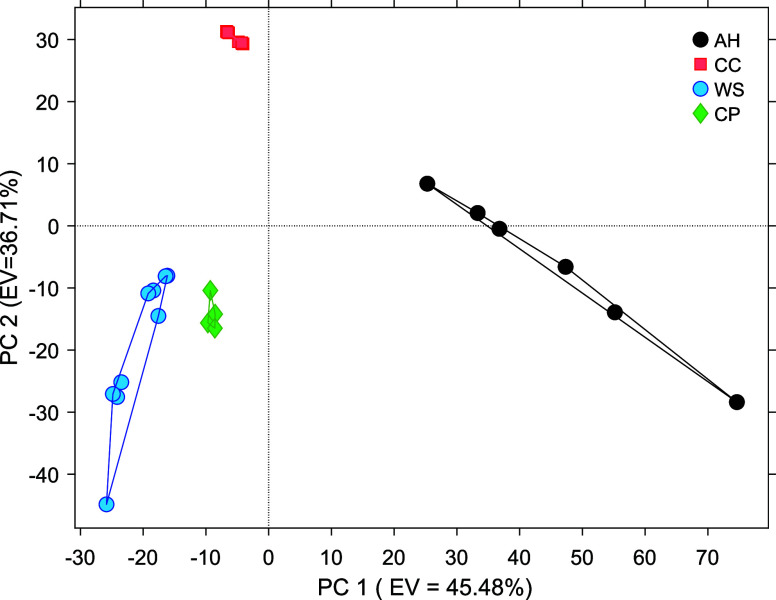
PCA score plot obtained from the voltammetric parameters of the gold (Au) sensor for different concrete conditions. Black circles: air humidity (AH); orange squares: carbonated concrete (CC); green diamonds: chloride-contaminated concrete (CP); blue circles: water-saturated concrete (WS).

In the PC1–PC2 space (Figure 15), measurements obtained under the same conditioning state cluster closely together, whereas different conditions form clearly separated groups. This clustering behavior indicates that the voltammetric response of the embedded sensor is reproducible within each condition and is systematically affected by the conditioning environment.

PC1 primarily captures variations in the overall current magnitude across the potential window. These variations can be associated with differences in the electrochemical environment of the pore network, particularly factors affecting mass transport and reactant availability such as moisture content and gas diffusion. In this regard, conditions characterized by water-saturated pores (WS and CP) tend to cluster on one side of the PC1 axis, while measurements obtained under atmospheric exposure (AH) appear on the opposite side. The cluster corresponding to the carbonated condition (CC) appears to be displaced relative to both extremes, reflecting a modified transport environment rather than a strictly intermediate state.

PC2 is more strongly influenced by variations in the electrical response of the system, which can be related to changes in the ionic transport within the pore solution. Samples exhibiting higher resistive behavior tend to be located at higher PC2 scores, whereas conditions associated with higher ionic conductivity appear at lower PC2 values. This trend is consistent with the expected influence of the pore-solution composition on the electrochemical response.

Overall, the separation of clusters in the PC1–PC2 space reflects systematic and physically meaningful differences in the electrochemical behavior of the system rather than random variability, supporting the suitability of the embedded sensor for discrimination between different concrete conditioning states.

To quantitatively support the clustering observed in the PCA score plot (Figure 14), Euclidean distances were calculated in the PCA space. For each conditioning state, the mean intraclass distance (Table 5), defined as the average distance of the measurements to their class centroid, was used as a measure of reproducibility. In addition, interclass centroid distances (Table 6) were calculated to assess the separation between different concrete conditions.

**Table 5:** Intraclass Mean Distances in the PC1–PC2 Space

Class	Intraclass mean distances (reproducibility)
AH	16.72
CC	1.41
WS	10.91
CP	2.06

**Table 6:** Interclass Centroid Distances in the PC1–PC2 Space

Class	Interclass centroid distances (discriminating ability)
AH–WS	67.33
AH–CC	63.13
AH–CP	54.95
CC–WS	52.27
CC–CP	44.75
CP–WS	12.85

The intraclass distances ranged from 1.41 (CC) to 16.72 (AH), while the interclass distances ranged from 12.85 (CP–WS) to 67.33 (AH–WS). The intraclass distances were substantially smaller than the interclass distances on average, indicating a clear separation between clusters despite limited overlap at the extremes. This quantitative analysis confirms that measurements obtained under the same conditioning state form compact clusters, whereas different concrete conditions are well separated in the PCA space.

## 4 Conclusions

The results demonstrate that the behavior of the embedded sensor in concrete is comparable to that observed in solution under similar pH conditions, exhibiting a similar voltammogram morphology. However, the peak intensities and potentials at which ion reactions occur between the pore solution and the sensor surface differ from those obtained in aqueous media. These variations arise from diffusion and ionic transport limitations within the hardened concrete matrix.

The redox process B/b retains the same electrochemical nature in both environments, although its behavior is influenced by the surrounding electrolyte. In solution, the response is quasi-reversible, governed by mixed diffusion–adsorption control, and characterized by a fast electron transfer (α*n* > 1). In concrete, however, the process exhibits predominantly diffusive control and slower kinetics (α*n* < 1), affected by diffusion resistance and ionic transport through the porous matrix.

Differences in Δ*E*_p_, *i*_B_/*i*_b_ and α*n*, together with the high *R*_u_ values, indicate that the limitations observed in concrete are primarily due to transport and internal polarization effects rather than the intrinsic kinetics of the electrochemical reaction.

Multivariate analysis (PCA) confirmed that the Au voltammetric sensor, when combined with electroanalytical techniques, enables clear discrimination among different concrete states and provides insight into the characteristics of the medium.

Overall, these findings demonstrate that Au voltammetric sensors can be effectively employed in porous materials containing interstitial water within their capillary networks, allowing in situ characterization of the internal electrolyte composition and monitoring of the material condition.
